# DDIT4 S‐Nitrosylation Aids p38‐MAPK Signaling Complex Assembly to Promote Hepatic Reactive Oxygen Species Production

**DOI:** 10.1002/advs.202201652

**Published:** 2022-05-05

**Authors:** Zilong Li, Qianwen Zhao, Yunjie Lu, Yangxi Zh

*Adv. Sci*. **2021**, *8*, 2101957

DOI: 10.1002/advs.202101957

**Figure 4:**
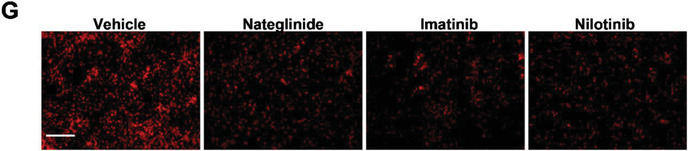
Imatinib, Nilotinib, and Nateglinide modulate ROS production and p38‐MAPK signaling by influencing DDIT4 S‐nitrosylation in vitro. g) Frozen sections were stained with DHE. h) Plasma ALT and AST levels.

In the originally published version of this article there is a wrong image for “Imatinib” was used in Figure 4g. The correct Figure 4g is reproduced below. The authors apologize for any inconvenience this may have caused.

